# Supplementary figures and images for: Minocycline decreases CCR2-positive monocytes in the retina and ameliorates photoreceptor degeneration in a mouse model of retinitis pigmentosa
Source: PLoS One. 2021 Apr 22;16(4):e0239108. doi: 10.1371/journal.pone.0239108 (PMC8062037; doi:10.1371/journal.pone.0239108)

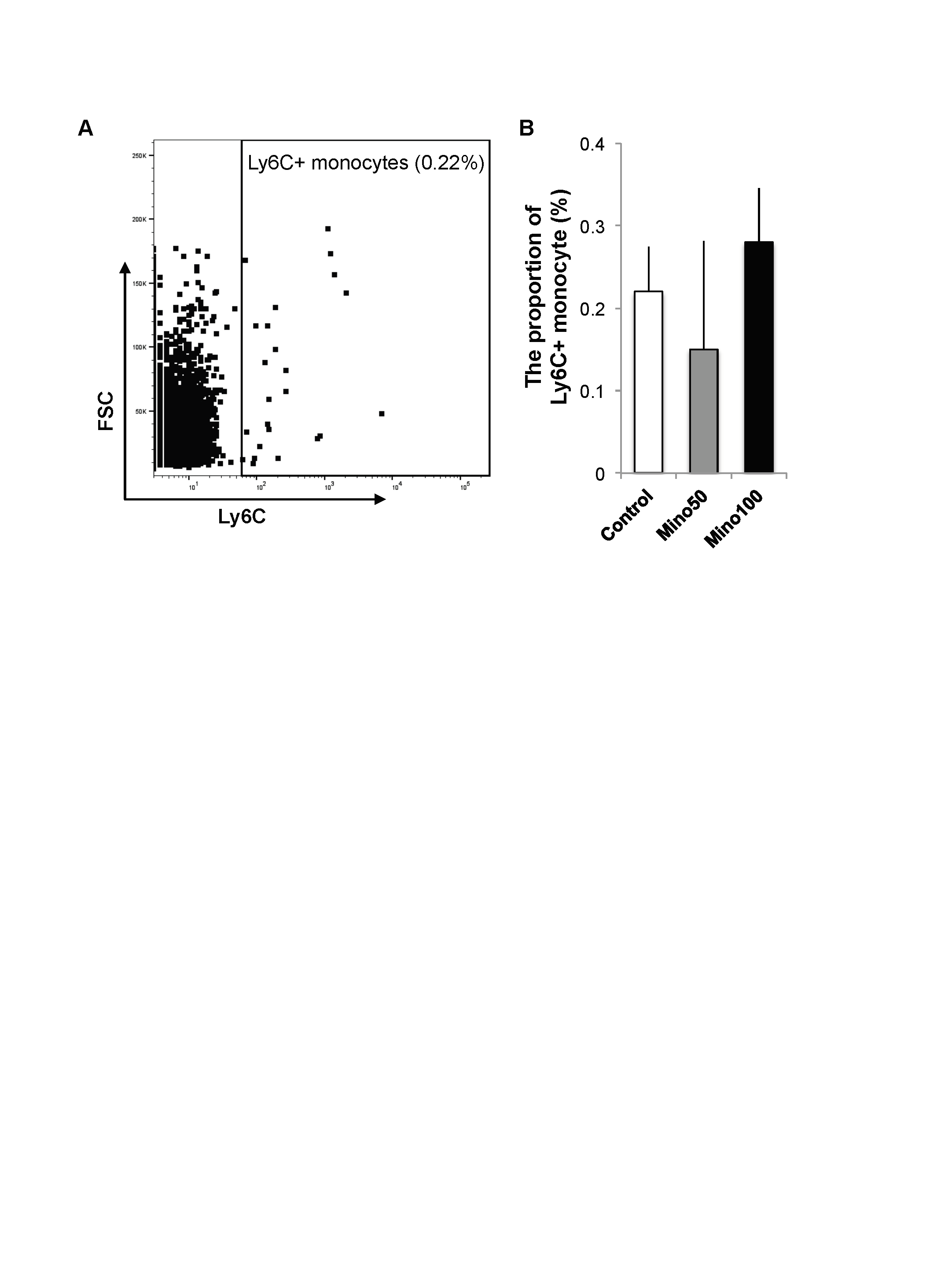

Supplement: S2 Fig — (A) Ly6C positive monocytes were detected in control Mertk−/−Cx3cr1GFP/+Ccr2RFP/+ mice by flow cytometry analysis. (B) The proportion of Ly6C positive monocytes in the control, Mino50, and Mino100 groups (n = 3 per group). (TIFF) [file pone.0239108.s002.tiff]
