# Supplementary material for: Minocycline decreases CCR2-positive monocytes in the retina and ameliorates photoreceptor degeneration in a mouse model of retinitis pigmentosa
Source: PLoS One. 2021 Apr 22;16(4):e0239108. doi: 10.1371/journal.pone.0239108 (PMC8062037; doi:10.1371/journal.pone.0239108)
Supplement: Attachment — Submitted filename: Response to reviewer.docx [file pone.0239108.s003.docx]

Alfred S Lewin, Ph.D.
Academic Editor

*PLOS ONE*

2021 Feb 26

Dear Dr. Lewin

Thank you for your e-mail on 2020 Oct 6th regarding our manuscript "Minocycline decreases CCR2-positive monocytes in the retina and ameliorates photoreceptor degeneration in a mouse model of retinitis pigmentosa" PONE-D-20-26780. In accord with the reviewers’ comment, we had carefully revised the manuscript. One by one responses to the reviewers are in the following pages. Thank you for taking time on our manuscript; we hope that our manuscript is now acceptable for publication in the PLOS ONE.

By the revision, figures had been re-organized as follows;

| **Original** | **Revised** | |  |
| --- | --- | --- | --- |
| Fig. 1 | Fig. 1 | | Images with only DAPI staining were removed. |
| Fig. 2 | Fig. 2 | | Pie charts were removed. |
| Fig. 3 | Fig. 3 | | Pie charts were removed. |
| Fig. 4 | Fig. 5 | | Results of Minocycline 100 mg/kg and TUNEL staining were added. |
| **Newly added figures** | | | |
| Fig. 4 | | The results of substrate based immunohistochemistry for CCR2-RFP positive cells were shown. | |
| Fig. 6 | | The neuroprotective effects of long-term minocycline treatment were shown. | |
| Suppl. Fig. 2 | | Ly6C-positive circulating monocytes observed by a flow cytometry were shown. | |

Best wishes,

Hideo Kohno

Response to Reviewers

Reviewer #1:

*1. Title: The current title is ambiguous and can benefit from rephrasing. Both “Ccr2 suppression” and “Cx3cr1/Ccr2-visualized” terms are imprecise and hard to understand. I suggest stating the main conclusions in the title: “Minocycline administration diminishes CCR2-positive monocyte number in the retina and ameliorates photoreceptor degeneration in a mouse model of retinitis pigmentosa”.*

Thank you for the comment, and in accord with the reviewer’s advice, we had revised the title of the manuscript.

*2. Abstract: The terms here and elsewhere relating to the suppression of Ccr2 expression are imprecise. What the authors document here is a decrease in the prevalence of CCR2-RFP+ cells in the outer retina and RPE layers with minocycline. Whether this finding resulted from the decreased recruitment of CCR2-expressing monocytes versus the actual suppression of CCR2 expression in monocytes resulting in a decrease in RFP-fluorescence is not investigated. The authors may want to reconsider the use of terms that indicate that CCR2 expression is decreased in individual monocytes from minocycline’s action (like “CCR2 suppression” ). The authors should pay attention to the CCR2 vs. Ccr2 case nomenclature throughout the manuscript to make sure the correct term is used for gene and protein names. The abstract can probably be shortened with the omission of methodological detail.*

Thank you for the comment. We agree that the terms relating the suppression of Ccr2 expression were not appropriate since, as the reviewer pointed out, we did not investigate whether the decrease of RFP-fluorescence was resulted from the decreased recruitment of CCR2-expressing monocytes or the actual suppression of CCR2 expression in monocytes. We had changed the wording to “decrease of the number of CCR2-RFP+ cells”. We apologize the usage of terms for gene and protein names was not paid attention. We had revised the terms in the whole manuscript. We removed the sentences describing methodology from the abstract.

*3. Results: It is important for the authors to establish what is being referred to as Cx3cr1-positive, Ccr2-positive and Cx3cr1/Ccr2 dual-positive cells are indeed GFP+/RFP-, GFP-/RFP+, and GFP+/RFP+ cells respectively. I had been looking throughout the manuscript for a confirmatory statement of this but I was not able to find it. The confusion is increased in Fig 2 and 3 in which the numbers in A-4, B-4, and C-4 do not match corresponding numbers in F, G, and H; in F, G, and H; dual-positive cells constitute the smallest constituency of all 3 cellular groups for all 3 conditions but in the pie charts, they consistently outnumber CCR2-postive cells. Without harmonizing this confusion, it is not possible to fully understand the data.*

We apologize the confusing figures in Fig. 2 and Fig. 3. In the pie charts; A-4, B-4, and C-4 (in the original figure), “Cx3cr2” and “Ccr2” represent GFP and FRP single positive cells, respectively (Cx3cr2 = GFP+RFP- and Ccr2 = GFP-RFP+). On the other hand, the panels D and E (in revised figure) indicate cell number of the sum of single positive and double positive cells; Cx3cr1 represents the sum of GFP+RFP- and GFP+RFP+, and Ccr2 represents the sum of GFP-RFP+ and GFP+RFP+. We found that information of the pie charts and the bar graphs are redundant. Therefore, we had removed the pie chart from the revised version. Related sentences had been revised and labelling of the graph and legend to the Fig 2 and Fig. 3 had been modified accordingly.

*4. Results: The ameliorative effect of minocycline on ONL thickness is actually quite minor and localized, leading one to wonder if it can be validated if more experimental numbers are accumulated. As such, the reference to this difference as “rescue” may be wording it too strongly. For smaller effects like this, more evidence is often desired to strengthen the conclusion, these include: (a) a dose dependent effect (i.e. more amelioration with higher doses of minocycline, like 100mg), (b) independent method of ascertaining ONL thickness (e.g. with in vivo  OCT measurements), and (c) other measurements of PR death (e.g. TUNEL labeling of apoptotic cells, length of outer segments, counting cone density etc). If these are available, they can help strengthen the conclusion that minocycline confers protection in this model.*

In accord with the reviewer’s suggestion, we had conducted additional experiments. We increased sample numbers and added samples treated with higher dose (100 mg/kg) of minocycline. In total, we examined 6 eyes for each condition. We found that the 50 mg/kg and 100 mg/kg minocycline treated mice showed thicker ONL than those of control mice; however the difference between 50 and 100 mg/kg doses was not statistically significant. We added results of 100 mg dose treated mice to Fig. 5A, B, C, and E, and related sentences had been modified. Regarding the wording “rescue”, we agree with the reviewer’s opinion that the wording is too strong, and revised the sentences.

*5. Discussion: The authors can focus more on interpreting the main findings of the manuscript, asking the questions of (a) whether minocycline inhibits infiltration of CCR2-expressing monocytes into the outer retina, or whether CCR2+ monocytes are converted into dual positive cells, to CX3CR1+, CCR2- cells, or even to CX3CR1-, CCR2- cells (which may render them undetectable in this assay) and (b) whether this effect of minocycline on CCR2-expressing monocytes is causally connected with the amelioration of PR degeneration, and if so, by what putative mechanisms. These discussion points follow directly from the main results and should constitute the main content of the discussion.*

Thank you for the comments, and we appreciate the reviewer to list the points of discussion. We agree with the reviewer that the discussion part should focus more on interpreting the main findings of the manuscript, and revised the whole part of the discussion

*Reviewer #2:*

*Comment #1*

*One incertainty is that they only use fluorescent markers to identify the cell types although subretinal macrophages (whatever type, microglial or monocyte derived) are known to become very autofluorescent as they phagocyte visual pigment rich outer segments of the photoreceptors. In any of these studies it would therefore be important that other techniques are used to corroborate the results using non-fluorescent techniques, such as substrate based immunohistochemistry (e.g. Fast red, if there is an antibody that differentiates GFP and RFP that should be easy), or in this case a difference in CCR2 expression by RT-PCR (or other Monocyte markers Ly6C etc…).*

In accord with the reviewer’s suggestion, we employed diaminobenzidine based detection protocol to detect CCR2-RFP positive cells by immunostaining. As expected, we observed stained cells in the subretinal space, supporting the idea that the signals observed by fluorescent were indeed RFP signals and not auto fluorescent. We added the results as Fig. 4. Related sentences had been revised. We also performed RT-qPCR; however, the results were unstable and did not give statistically significant results, therefore we did not include the RT-qPCR results.

*Comment #2 Protective effect of Minocycline: The group has previously shown that CCL2 and CCL3 deletion very significantly preserves photoreceptors in 8 week old Mertk-/- mice. In their 2014 paper control mice were left with less than 15µm of ONL in Mertk-/- mice. Here at the same age we have more than 30µm in controls and the minocycline treated mice are not all that different. To be convincing I think a longer treatment and a later stage in the degeneration with a bigger difference would help…*

We agree that there was difference in ONL thickness in 8-week retina between the current study and our previous study, and have currently no rational explanation regarding the discrepancy. In accord with the reviewer’s suggestion, we examined effects of longer treatment of minocycline in *Mertk* knockout mice. We used 8-weeks mice as starting point, and after 8 weeks, we harvested the mice. We observed effects of minocycline to keep thickness of the ONL in this condition, but the degree of the difference of thickness of ONL between control and minocycline treated mice was similar with that observed in short-term minocycline treatment (from 6 to 8 weeks of age). However, the results supported the idea that the longer and later treatment of minocycline is also effective to reduce the degeneration. We added these results into Fig. 6, and related sentences were modified and added.

*Comment #3 Mechanism: finally it would be nice to have a little more insight in the mechanism. Does minocycline change the expression levels of CCL2 and CCL12, the two main ligands of CCR2 in mice? Does it change the populations of circulating monocytes? Or does it affect immunosupressivity in the eye? Some of these questions should be very easy to get answers to and would strengthen the paper.*

Thank you for the valuable comments, and we examined the expression of these genes including immunosupressive cytokine genes by RT-qPCR, but we did not observe significant changes by the treatment of minocycline. We also examined peripheral circulating monocyte by a flow cytometer, and the population of the monocyte was not changed by the minocycline. We added monocyte results as supplemental Fig. 2, and related sentences had been revised accordingly.
